# Characterization of Rhesus Macaque Liver-Resident CD49a^+^ NK Cells During Retrovirus Infections

**DOI:** 10.3389/fimmu.2020.01676

**Published:** 2020-07-31

**Authors:** Daniel R. Ram, Christian F. Arias, Kyle Kroll, Brady Hueber, Cordelia Manickam, Rhianna A. Jones, Scott T. Smith, Spandan V. Shah, Valerie H. Varner, R. Keith Reeves

**Affiliations:** ^1^Center for Virology and Vaccine Research, Beth Israel Deaconess Medical Center, Harvard Medical School, Boston, MA, United States; ^2^Boston University School of Medicine, Boston, MA, United States; ^3^Ragon Institute of Massachusetts General Hospital, MIT, and Harvard, Cambridge, MA, United States

**Keywords:** natural killer, HIV, non-human primate, macaque, SIV

## Abstract

CD49a^+^ tissue resident NK cells have been implicated in memory-like NK cell responses, but while this population is well-characterized in mice and in humans, they are poorly described in non-human primates (NHP) which are particularly critical for modeling human viral infections. Others and we have shown that memory-like NK cells are enriched in the liver and because of the importance of NHP in modeling HIV infection, understanding the immunobiology of CD49a^+^ NK cells in SIV-infected rhesus macaques is critical to explore the role of this cell type in retroviral infections. In this study mononuclear cells isolated from livers, spleens, and peripheral whole blood were analyzed in acutely and chronically lentivirus-infected and experimentally-naïve Indian rhesus macaques (RM). NK cells were then identified as CD45^+^CD14^−^CD20^−^CD3^−^NKG2A/C^+^ cells and characterized using multiparametric flow-cytometry. Our data show that in RM, CD49a^+^ NK cells increase in the liver following retroviral infections [median = 5.2% (naïve) vs. median = 9.48% (SIV+) or median = 16.8% (SHIV+)]. In contrast, there is little change in CD49a^+^ NK frequencies in whole blood or spleens of matched animals. In agreement with human and murine data we also observed that CD49a^+^ NK cells were predominantly Eomes^low^ T-bet^low^, though these frequencies are elevated in infected animal cohorts. Functionally, our data suggests that infection alters TNF-α, IFN-γ, and CD107a expression in stimulated CD49a^+^ NK cells. Specifically, our analyses found a decrease in CD49a^+^ CD107a^+^ TNFα^+^ IFNγ^−^ NK cells, with a simultaneous increase in CD49a^+^ CD107a^+^ TNFα^−^ IFNγ^+^ NK cells and the non-responsive CD49a^+^ CD107a^−^ TNFα^−^ IFNγ^−^ NK cell population following infection, suggesting both pathogenic and inflammatory changes in the NK cell functional profile. Our data also identified significant global differences in polyfunctionality between CD49a^+^ NK cells in the naïve and chronic (SHIV+) cohorts. Our work provides the first characterization of CD49a^+^ NK cells in tissues from RM. The significant similarities between CD49a^+^ NK cells from RM and what is reported from human samples justifies the importance of studying CD49a^+^ NK cells in this species to support preclinical animal model research.

## Introduction

Natural killer (NK) cells are considered as the prototypic innate immune effector cell capable of rapid and broad (non-specific) responses to several agents—including viral infections and cancerous cells. NK cells are generally thought to function through engagement of either activating or inhibitory molecules on the cell surface, leading to activation, or repression of NK cell function depending on the ratio of receptor engagement (1–3). Recently, NK cells have been identified as also having peptide-specificity and memory-recall potential, once previously thought to belong only to adaptive immune cells, like B cells or T cells (4–6). Adaptive NK cells have been shown to be enriched in the livers of mice (7) and non-human primates (NHP) (5), and recently in human livers from BLT mice (8).

The α1β1 integrin CD49a (also VLA-1) has been shown to be associated with liver-resident lymphocytes and is further described as one of several markers for adaptive NK cells that accumulate in the liver (7, 9, 10). CD49a expression on uterine NK cells (uNK) and other tissue-resident NK cells, may also delineate adaptive-like properties (11–13). CD49a may play a functional role in NK cell responses in tissues by regulating migration, or perhaps influencing proliferation in the tissues (14). In humans it has been shown that CD49a^+^ NK cells are enriched in liver cirrhosis and further that CD49a^+^CD25^+^ NK cells have enhanced proliferative capacity *ex vivo* (15). Further, ligation of CD49a has been shown to influence tyrosine kinase signaling leading to IL-2 dependent NK cell activation (16). CD49a has been shown to have many binding partners, but is predominantly thought to interact with collagens (I, IV, IX, and XVI) (17–19) and laminins (111 and 112) (20). Additionally, CD49a has been shown to interact with Galectins 1, 3, and 8 (21, 22) and semaphorin 7A (23), which has been implicated in cytokine-induced NK cell memory responses (24).

In contrast, CD49b^+^ (DX5 in mice, also α2β1) NK cells have been characterized as more migratory, and show greater similarity to conventional spleen NK cells in mice (11, 25), providing a more direct comparison for tissue-resident vs. trafficking NK cells. CD49b may also play a role in binding the complement molecule C1q, although whether this occurs in NK cells is still unclear (26). Recent mouse studies have shown that CD49b is not required for NK cell effector responses in the spleen or liver, but may play a role in the proliferation of NK cells in response to ectromelia virus (ECTV) and mouse CMV (MCMV) infection (27). The role of CD49b on human NK cells is not as clear, though it likely also plays a role in NK cell migration (28).

While there have been several studies characterizing CD49a^+^ NK cells in mice, humanized mice, and humans, to date these cells remain unexplored in NHP. Given the role that NHP play for modeling several human diseases, like HIV/AIDS, ZIKA, influenza, and tuberculosis (29–36), it is critical to characterize this population of NK cells in relevant NHP models.

## Materials and Methods

### Ethics Statement

All animals were housed at Biomere Inc. (Worcester, MA) or the New England Primate Research Center (Southborough, MA). All study blood samplings were reviewed and approved by the local Institutional Animal Care and Use Committee. All animal housing and studies were carried out in accordance with recommendations detailed in the Guide for the Care and Use of Laboratory Animals of the National Institutes of Health with recommendations of the Weatherall report; “The use of non-human primates in research.” Animals were fed standard monkey chow diet supplemented daily with fruit and vegetables and water *ad libitum*. Social enrichment was delivered and overseen by veterinary staff and overall animal health was monitored daily. Animals showing significant signs of weight loss, disease, or distress were evaluated clinically and then provided dietary supplementation, analgesics, and/or therapeutics as necessary. Animals were euthanized with an overdose of pentobarbital, followed by necropsy. Liver and spleen samples were then processed as detailed below.

### Animals

Samples from sixteen necropsied Indian origin rhesus macaques (*Macaca mulatta*) were analyzed in this study: four experimentally naïve animals, seven animals that were infected with SIV_mac251_/SIV_mac239_ for 7–14 days, and five chronically infected with SHIVSF162P3. All experiments were performed with approval from the local Institutional Animal Care and Use Committee (IACUC). All animals were group housed until the start of the study and then infected animals were housed under BSL2 conditions.

### Macaque Samples

Liver and spleen mononuclear cells were isolated using standard isolation protocols (5). Briefly, after *ex vivo* excision the liver was flushed and then liver mononuclear cells were isolated using mechanical disruption followed by density-gradient centrifugation layered over 60% Percoll. Splenic mononuclear cells were isolated by mechanical disruption. Contaminating red blood cells were lysed using an ACK lysis buffer (Gibco, Cat. No. A1049201). Cell aliquots were immediately cryopreserved in 90% FBS, 10% DMSO (Sigma) and stored in liquid nitrogen vapor. Whole blood samples were collected in EDTA blood collection tubes and following lysis of red blood cells an aliquot was immediately used for flow cytometry analysis.

### Functional Assay

Cryopreserved liver and spleen mononuclear cells were cultured in R10 medium (RPMI + 10% FBS) only or stimulated with phorbol myristate acetate (PMA, 2.2 μg/mL, Sigma) and Ionomycin (5 μg/mL, Sigma) for 4 h in the presence of monensin (GolgiStop) and Brefeldin A (GolgiPlug; BD Biosciences, concentrations as recommended by manufacturer). Cells were then processed for flow cytometry.

### Flow Cytometry

All antibodies were purchased from BD Biosciences unless specified otherwise and their clone information is in parentheses. For the liver phenotypic panel, antibodies against the following cell antigens were used: Eomes-FITC (WD1928, Life Technologies), CD150-BB630 (A12), CD195-BB700 (3A9), SYK-BB790 (4D10), CD49a-PE (SR84), CD49b-PECF594 (AK-7), CD49e (NKI-SAM1, Biolegend), CD336-PE Cy5 (Z231, Beckman Coulter), CD20-PE Cy5.5 (2H7, Life Technologies), T-bet-PE Cy7 (4B10, Life Technologies), DAP12-Alexa405 (405288, Novus), CD69-BV510 (FN50, Biolegend), CD14-BV570 (M5E2, Biolegend), CD337-BV605 (p30-15), CD366-BV650 (F38-2E2, Biolegend), PD-1-BV750 (EH12.1), Zap70-BV786 (1E7.2), CD3-BUV395 (SP34.2), CD16-BUV496 (3G8), CD8α-BUV563 (RPA-T8), CD45-BUV615 (D058-1283), HLA-DR-BUV661 (G46-6), CD56-BUV737 (NCAM16.2), CD62L-BUV805 (SK11), CD159a-APC (Z199, Beckman Coulter), FcεRI-A700 (rabbit polyclonal, Millipore, conjugated in-house). For the liver functional panel antibodies against the following cell antigens were used: CD45-FITC (D058-1283), CD49a-PE (SR84), CD49b-PECF594 (AK-7), CD159a-PE Cy7 (Z199, Beckman Coulter), CD3-BV450 (SP34.2), TNF-α-BV650 (MAb11), IFN-γ-BV711 (B27), CD107a-BV786 (H4A3), CD20-BUV395 (L27), CD16-BUV496 (3G8), CD56-BUV563 (NCAM16.2), CD14-BUV737 (MφP9), HLA-DR-Alexa700 (G46-6), CD8α-APC Cy7 (SK1). Flow cytometry data was acquired on a BD LSRII or BD FACSymphony A5 (BD Biosciences, La Jolla, CA) and analyzed with FlowJo software (version 10.2, Tree Star, Ashland, OR).

For the spleen phenotypic panel, antibodies against the following cell antigens were used: Eomes-FITC, CD49a-PE, CD49b-PECF594, CD336-PERCP Cy5.5, CD3-V450, CD56-BV570, CD337-BV605, CD366-BV-650, CD14-BV711, CD45-BV786, CD20-BUV395, CD16-BUV496, CD159a-APC, HLA-DR-A700, and CD8α-APC Cy7. For the spleen functional assay, the antibodies used were against the following antigens: MIP-1β-FITC, CD49a-PE, Granzyme B-ECD, CD107a-PERCP Cy5.5, IFNγ-PE Cy7, CD3-V450, CD56-BV570, CD14-BV711, CD45-BV786, CD20-BUV395, CD16-BUV496, CD159a-APC, TNF-α-A700, and CD8α-APC Cy7. All antibody clones were consistent between spleen and liver samples. The spleen flow cytometry data was acquired on an LSRII (BD Biosciences, La Jolla, CA) and analyzed with FlowJo software (version 10.2, Tree Star, Ashland, OR).

### Statistical Analyses

Statistical and graphing analyses were performed with GraphPad Prism 8.0 software (GraphPad Software, La Jolla, CA). Non-parametric Mann-Whitney *U*-or Wilcoxon tests were used where indicated, and a *p-*value of *p* < 0.05 was considered to be statistically significant. Permutation analyses were carried out in SPICE (37) in order to compare the polyfunctional data plots.

## Results

### Frequencies of CD49a^+^ NK Cells Are Elevated Following Retroviral Infection

**Figure 1:**
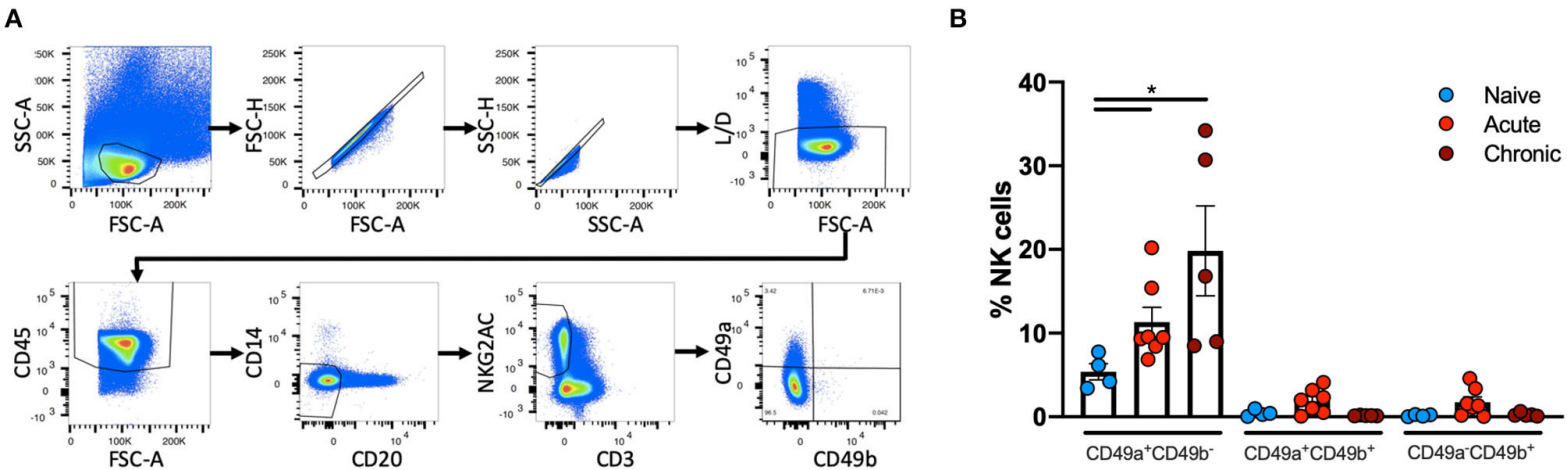
CD49a^+^ NK frequencies vary in in Liver following infection. **(A)**, Representative, gating strategy showing the identification of CD49a^±^CD49b^±^ NK cells. **(B)** Quantification of frequencies of CD49a^±^CD49b^±^ cells in livers of naïve (*n* = 4), acute SIV+ (*n* = 7), or chronic SHIV+ (*n* = 5) animals. Mann-Whitney *U-*test was used to determine statistical significance, *p < 0.05.

Liver NK cells from naïve, acute SIV-infected or chronically SHIV-infected rhesus macaques were identified using the following previously defined criteria: CD45^+^CD14^−^CD20^−^CD3^−^NKG2AC^+^ (38, 39). This co-expression analysis of NKG2A and NKG2C (CD159a and CD159c) identifies the majority of NK cells in rhesus macaque blood and tissues. These NK cells were then further characterized by the expression of CD49a and CD49b (Figure 1A). Quantification of CD49a^±^b^±^ NK cells revealed that the majority of NK cells in the liver did not express CD49a or CD49b, but interestingly there was a significant increase in the frequencies of CD49a^+^b^−^ NK cells from acute SIV+ (median = 9.48%) or chronic SHIV+ (median = 16.8%) infected animals as compared to the naïve group (median = 5.2%; Figure 1B). Statistical comparisons between acute and chronic infection groups are not shown for most analyses given the different challenge viruses. For a subgroup of SIV+ animals we had the opportunity to longitudinally monitor expression of CD49a and CD49b in the blood, and though we observed minor animal-to-animal variability we did not observe any significant changes in the frequencies of CD49a^+^ or CD49b^+^ NK cells over either 7 or 14 days following challenge with SIV (Supplementary Figure 1). We also assessed frequencies of CD49a and CD49b in the spleen and observed a reduction in CD49a^+^ NK cells in the chronic (SHIV+) cohort relative to naïve, albeit not statistically significant (Supplementary Figure 2). Liver resident NK cells also do not generally express CD49e, as this integrin is an indicator of cells in circulation (40). For this reason we also assessed CD49e on NK cells from the naïve and acute (SIV+) cohorts and found that, as expected, CD49a^+^ and CD49e^+^ NK cells were generally mutually exclusive (Supplementary Figure 3). Further, we observed that the frequency of CD49a^−^e^+^ NK cells showed a small but non-significant (*p* > 0.05) increase in livers of the acute (SIV+) infection cohort relative to the naïve group (Supplementary Figure 3).

### Liver Resident CD49a^+^ NK Cells Are Phenotypically Distinct

**Figure 2:**
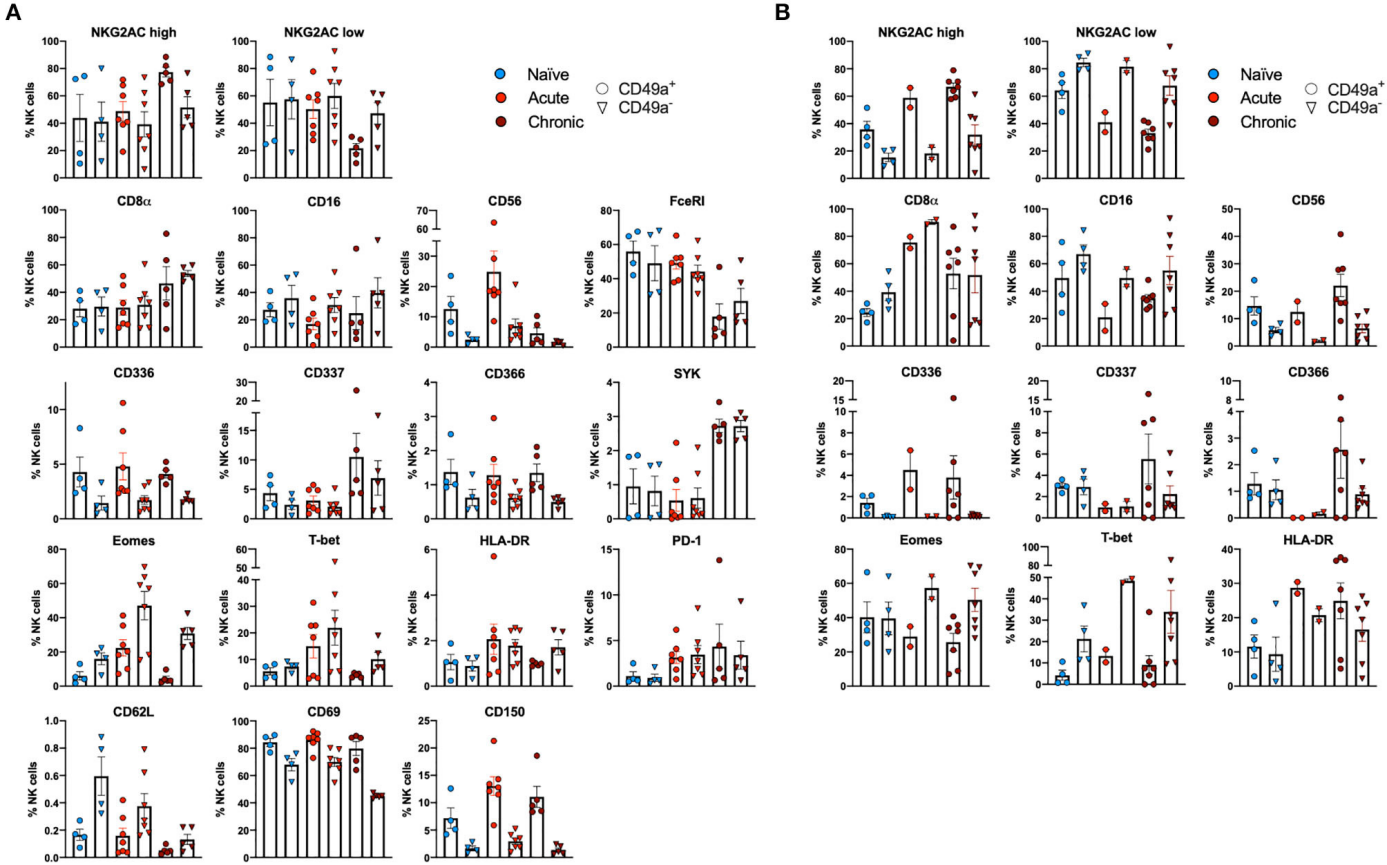
CD49a^+^ NK cells exhibit tissue-specific phenotype. **(A)** Phenotypic characterization of Liver CD49a^±^ NK cells from naïve macaques (*n* = 4) or macaques acutely infected with SIV (*n* = 6) or chronically infected with SHIV (*n* = 5). **(B)** Phenotypic characterization of Spleen CD49a^±^ NK cells from naïve macaques (*n* = 4) or macaques acutely infected with SIV (*n* = 2) or chronically infected with SHIV (*n* = 7). Mann-Whitney *U-*test or Wilcoxon test was used as indicated in Supplementary Data Sheets 1A,B to determine statistical significance.

**Figure 3:**
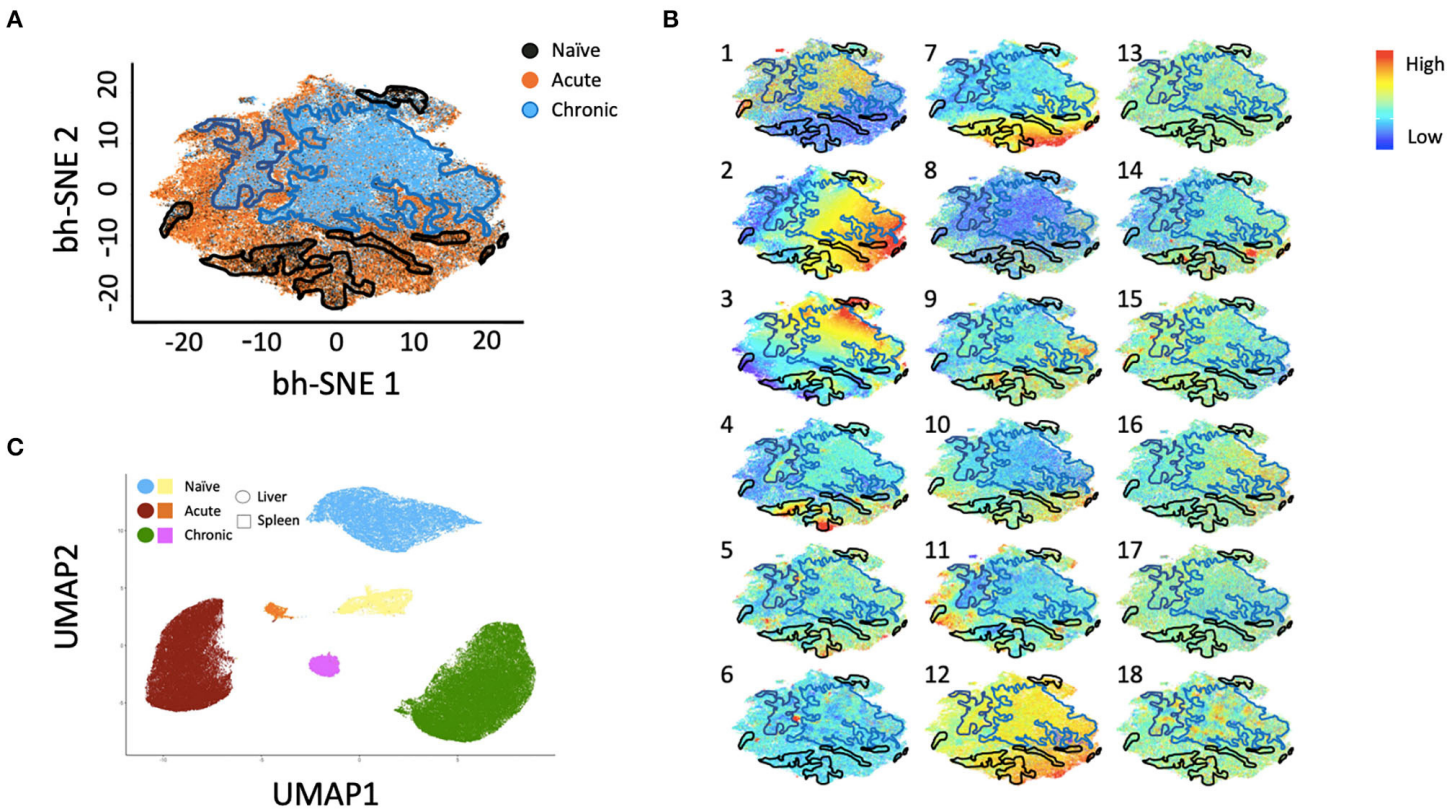
*bh*-SNE reveals infection-specific expression profiles in Liver CD49a^+^ NK cells. **(A)** *bh*-SNE representations illustrating the distribution of various phenotypic markers on CD49a^+^ NK cells from naïve (enclosed by black boundaries), chronic SHIV+ (enclosed by blue boundaries), or acute SIV+ samples (remaining unbound orange space). **(B)** Normalized expression of various phenotypic markers are superimposed on **(A)** showing distribution of: (1) CD8α, (2) CD159AC (NKG2AC), (3) CD49b, (4) Eomes, (5) T-bet, (6) PD-1, (7) FcεRI/FcRγ, (8) CD16, (9) CD337 (NKp30), (10) CD336 (NKp44), (11) CD150, (12) Dap12, (13) CD62L, (14) CD69, (15) CD195, (16) CD366 (Tim-3), (17) HLA-DR, and (18) Zap70. **(C)** UMAP clustering reveals relationships between liver and spleen CD49a^+^ NK cells.

Multiparametric flow cytometry analysis revealed several phenotypic changes following infection in CD49a^+^ NK cells from livers (Figure 2A) and spleens (Figure 2B). Interestingly, in livers we saw significant changes in several proteins, including altered frequencies Eomes, FcεRI, Syk, CD62L, and PD-1 NK cells in the infected groups relative to naïve (Figure 2A, Supplementary Data Sheet 1A). In the retrovirus-infected cohorts we also observed several changes (at or approaching *p* ≤ 0.05) between CD49a^+^ and CD49a^−^ NK cells, including CD16, CD56, CD62L, CD69, CD150, CD336 (NKp44), CD366 (Tim-3), Eomes, NKG2AC^high^, and NKG2AC^low^ and T-bet (Figure 2A, Supplementary Data Sheet 1A). Though the cell frequencies are low it is interesting to note elevated levels of CD336 (NKp44) on CD49a^+^ NK cells relative to CD49a^−^ NK in the infected cohorts as NKp44^+^ NK cells are potent antiviral effectors (41). It is well-established that the currently available antibodies to detect NKG2A cross react with NKG2C in NHP (42) and thus the convention is to term the cell population identified by the anti-NKG2A antibody as NKG2AC^+^. As a result, we have developed an RNA flow-based approach to discriminate between NKG2A and NKG2C (39, 43). However, some observations suggest that the NKG2AC^high^ population corresponds to a population that predominantly expresses NKG2A (relative to NKG2C), whereas the NKG2AC^low^ population corresponds predominantly NKG2C expressing cells (39)—shown in Supplementary Figure 4 where gene expression of KLRC1 (NKG2A) is elevated in the NKG2AC high population, whereas gene expression of KLRC2 (NKG2C) is elevated in the NKG2AC ^low^ population in peripheral blood mononuclear cells from both experimentally naïve animals (CMV+) and an acute SIV+ cohort (39). These observations thus suggest that in the livers of the chronic (SHIV+) group there is an elevation of NKG2A^+^ CD49a^+^ NK cells. Figures 2A,B also illustrate several differences between liver and spleen CD49a^+^ NK cells: elevated CD56 in liver CD49a^+^NK cells from the acute (SIV+) cohort relative to spleen, as well as elevated CD8α and HLA-DR in spleen CD49a^+^ and CD49b^+^ NK cells following infection relative to the liver. There were several significant changes in the CD49a^+^ vs. CD49a^−^ NK populations and these are highlighted in Supplementary Data Sheet 1B. We also utilized UMAP in order to assess the multiparametric relatedness of the various CD49a^+^ NK cell populations between liver and spleen, and in the naïve, acute (SIV+) and chronic (SHIV+) cohorts (Figure 3C). We observed that while the populations clustered into distinct groups, the spleen and liver samples appeared to generally localize according to infection status. Using *bh*-SNE we also observed the clearest overall phenotypic differences between naïve and chronic (SHIV+) samples as opposed to naïve and acute (SIV+) animals shown by the distinct clustering in the chronic (SHIV+) relative to naïve samples (Figure 3). Interestingly, there was a consolidation/reduction of distinct CD49a^+^ NK populations from the naïve samples (outlined in black) to a smaller number of clusters in both the acute (SIV+) and chronic (SHIV+) infected cohorts (outlined in orange and blue, respectively, Figure 3A). By overlaying the normalized expression of several phenotypic markers, we were able to see their relative expression on the naïve and infection cohorts (Figure 3B). There were several phenotypic markers that seemed to drive the overall differential clustering, including CD8α, CD16, CD56, NKG2A, CD366, CD337, CD336, T-bet, and Eomes.

### Liver CD49a^+^ NK Cells Display Preferential IFNγ Production During Infection

**Figure 4:**
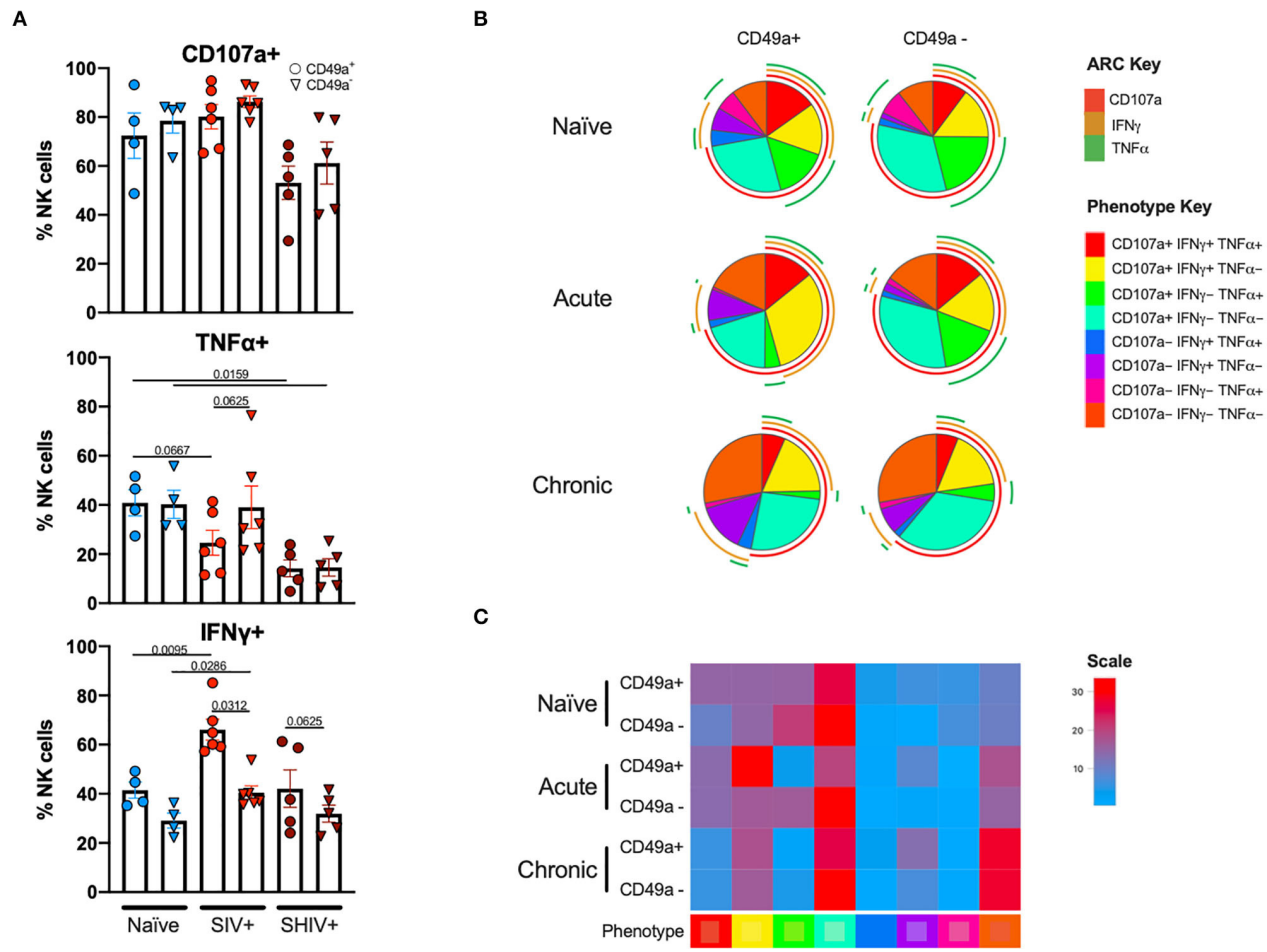
CD49a^+^ NK cells exhibit enhanced IFNγ during infection. **(A)** Bar graphs showing quantification of IFNγ, TNFα, and CD107a expression following 4 h stimulation with PMA and ionomycin in liver CD49a^±^ NK cells from naïve macaques (*n* = 4) or macaques acutely infected with SIV (*n* = 6) or chronically infected with SHIV (*n* = 5). **(B)** Pie charts illustrating the proportion of NK cells that exhibit polyfunctional characteristics following 4 h stimulation with PMA and ionomycin. **(C)** Heatmap showing comparison of polyfunctional phenotype across the infection groups. The scale shows population frequencies of NK cells. Mann-Whitney *U-*test or Wilcoxon test was used to determine statistical significance.

Following stimulation of liver mononuclear cells, CD49a^+^ NK cells upregulated CD107a and production of TNFα and IFNγ (Figure 4). Interestingly, relative to the naïve cohort, cells from the chronic SHIV+ cohort produced reduced levels of TNFα (*p* = 0.016; Figure 4A). TNFα levels were also reduced in the acute SIV+ cohort relative to naïve (*p* = 0.067). While all groups showed elevated frequencies of IFNγ in CD49a^+^ relative to CD49a^−^ NK cells, the differences were most robust in retrovirus-infected cohorts. We also assessed functional properties of spleen CD49a^+^ NK cells in naïve, acute (SIV+) and chronic (SHIV+) and observed significant elevation of IFNγ, Granzyme B (GZB), and MIP1β in CD49a^+^ vs. CD49a^−^ NK cells in the chronic infection cohort (Supplementary Figure 5). Analysis of polyfunctionality in liver samples revealed a significant loss of CD49a^+^ CD107a^+^ IFNγ^−^ TNFα^+^ NK cells following retroviral infection, whereas there was a significant increase in the CD49a^+^ CD107a^+^ IFNγ^+^ TNFα^−^ only in the acute SIV+ cohort (Figures 4B,C). Further, we observed an increase in the CD49a^+^ CD107a^−^ TNFα^−^ IFNγ^−^ population in the chronic infection group relative to naïve animals. In order to compare the various polyfunctional populations we also carried out a permutation test with 1,000,000 permutations. This analysis revealed significant differences between naïve and chronic (SHIV+) CD49a^+^ NK cells (*p* = 0.0492, Supplementary Data Sheet 1C).

## Discussion

CD49a^+^ NK cells are still poorly characterized in any tissue from NHP. Given their association with liver-residence and that liver-resident CD49a^+^ NK cells have been thought to play a role in the adaptive NK cell response (5, 7, 9, 10), understanding CD49a^+^ NK cells may provide a potential novel avenue for vaccine or immunotherapy design. It is therefore crucial to evaluate the impact of HIV and SIV infections on this population. In human livers CD49a^+^ NK cells have been shown to also express high levels of NKG2C (10). Here we also show elevation of NKG2AC^high^ CD49a^+^ NK cells following chronic infection (with SHIV). This suggests that the resulting NK cells may possess greater inhibitory properties, since NKG2A is an inhibitory molecule and has been suggested to play a role in diminution of the NK response in the liver of humanized mice (44). This may provide an opportunity for NKG2A blockade therapy in order to restore NK cell function (44, 45). The concurrent observation of increased frequencies of the putatively non-functional CD49a^+^CD107a^−^TNFα^−^IFNγ^−^ NK cell following retroviral infection may suggest a diminished NK cell response following retroviral infection that may be different from what is seen in human livers, albeit in the context of cancer (10). While the expansion of the polyfunctional CD49a^+^CD107a^+^IFNγ^+^TNFα^−^ population was not necessarily surprising, given the role of IFNγ in antiviral responses, it was surprising to see a loss of the CD49a^+^ CD107a^+^ IFNγ^−^ TNFα^+^ population in CD49a^+^ NK cells from the acute (SIV+) cohort and both CD49a^+^ and CD49a^−^ NK cells in the chronic (SHIV+) cohort. It is unclear why the frequency of CD107a^+^ IFNγ^−^ TNFα^+^ polyfunctional NK cells were unchanged in the CD49a^−^ NK cells from acute (SIV+) cohort as compared to the naïve cohort. Whether or not acute or chronic infection result in altered responses requires further investigation as this study was not designed to specifically resolve this possibility.

Our phenotypic characterization has also highlighted several populations of interest, including CD49a^+^ NK cells expressing CD56 or CD150 (SLAM) during acute or chronic retrovirus infections. While the expression of certain proteins like Eomes and T-bet appear low in our naïve samples, overall the ranges fall within observed values from our work and from others as well (5, 8, 46). We also see several differences in CD49a^+^ NK cells between the liver and the spleen, particularly in their differential expression of HLA-DR following retroviral infection. The role of HLA-DR on NK cells is still unclear. HLA-DR expression has been posited as a marker of NK cell activation but it has also been shown to play a role in immune modulation (47), though it has also been suggested that while HLA-DR+ NK cells are less phenotypically mature they still display high functional activity (48). While we did not see a statistically significant increase in frequencies of CD49a^−^e^+^ NK cells in the livers of acute SIV-infected macaques relative to naïve animals, the small increasing trend may be interesting to explore in further studies with a larger animal cohort. Regardless, our multiparametric phenotypic and functional characterization of CD49a^+^ NK cells provides the first investigation of CD49a^+^ NK cells in livers of both naïve and infected RM cohorts. Understanding how CD49a^+^NK cells are modulated in the liver following infection may provide clues to how we can best engage this liver-resident NK cell population and possibly improve responses to SIV/HIV infections.

## Data Availability Statement

All datasets presented in this study are included in the article/Supplementary Material.

## Ethics Statement

This animal study was reviewed and approved by Harvard IACUC and Biomere IACUC.

## Author Contributions

DR and RR conceived the experiment and wrote the manuscript. DR, CA, KK, BH, CM, RJ, STS, SVS, and VV carried out experiments. DR, CA, KK, and RR analyzed the data. All authors contributed to the article and approved the submitted version.

## Conflict of Interest

The authors declare that the research was conducted in the absence of any commercial or financial relationships that could be construed as a potential conflict of interest.

## Supplementary Material

Supplementary Figure 1 Frequencies of peripheral blood CD49a^+^ NK cells are unchanged during acute SIV infection. **(A)** Frequencies of NK cells in whole blood of acute-SIV infected animals. Frequencies of **(B)** CD49a^+^ or **(C)** CD49b^+^ NK cells in the whole blood following acute SIV infection.

Supplementary Figure 2 Frequencies of CD49a^±^b^±^ in spleens. Quantification of frequencies of CD49a^±^CD49b^±^ cells in spleens of naïve (*n* = 4), acute SIV+ (*n* = 2), or chronic SHIV+ (*n* = 7) animals. Mann-Whitney *U-*test was used to determine statistical significance, ^*^*p* ≤ 0.05.

Supplementary Figure 3 Frequencies of CD49a^±^e^±^ NK cells in livers. **(A)** Representative flow plot showing identification of CD49a^±^e^±^ NK cells from liver samples. **(B)** Quantification of CD49a^±^e^±^ NK cell populations from livers of naïve (*n* = 3) and acute SIV+ (*n* = 3) animals.

Supplementary Figure 4 NKG2AC^high^ and NKG2AC^low^ populations exhibit unique KLRC1 and KLRC2 gene expression patterns. **(A)** Gating strategy showing identification of gene expression of KLRC1 and KLRC2 in NKG2AC high and NKG2AC low populations. **(B)** Quantification of KLRC1^+^KLRC2^+^ (K1+K2+) and KLRC1^−^KLRC2^+^ (K1–K2+) populations in NKG2AC^high^ (H) or NKG2AC^low^ (L) NK cells from CMV+ and SIV-infected animals as per (39). Mann-Whitney *U-*test was used to determine statistical significance, ^*^*p* < 0.05, ^**^*p* < 0.01.

Supplementary Figure 5 Functional characterization of spleen CD49a^+^ NK cells. **(A)** Bar graphs showing quantification of CD107a, TNFα, and IFNγ expression in spleen CD49a^±^ NK cells from naïve macaques (*n* = 2) or macaques acutely infected with SIV (*n* = 2) or chronically infected with SHIV (*n* = 5). Wilcoxon test was used to determine statistical significance, ^*^*p* < 0.05.

Supplementary Data Sheet 1 Statistics tables. *p*-value tables showing comparisons in **(A)** liver and **(B)** spleen phenotype data (from Figure 2) as well as **(C)** permutation testing of polyfunctional populations (from Figure 4B). For **(A,B)** Wilcox test was used when comparing CD49a^+^ and CD49a^−^from the same infection group whereas Mann-Whitney *U*-test was used when comparing across infection cohorts. *p* ≤ 0.05 is shown by bold font. 1,000,000 iterations were used in order to carry out the permutation test in **(C)**. *p* ≤ 0.05 is shown in cells highlighted in pink as generated by SPICE (37). Statistical comparisons between acute and chronic infection groups are not shown for these analyses given the different challenge viruses.
