# Supplementary figures and images for: Characterization of Rhesus Macaque Liver-Resident CD49a+ NK Cells During Retrovirus Infections
Source: Front Immunol. 2020 Jul 31;11:1676. doi: 10.3389/fimmu.2020.01676 (PMC7411078; doi:10.3389/fimmu.2020.01676)

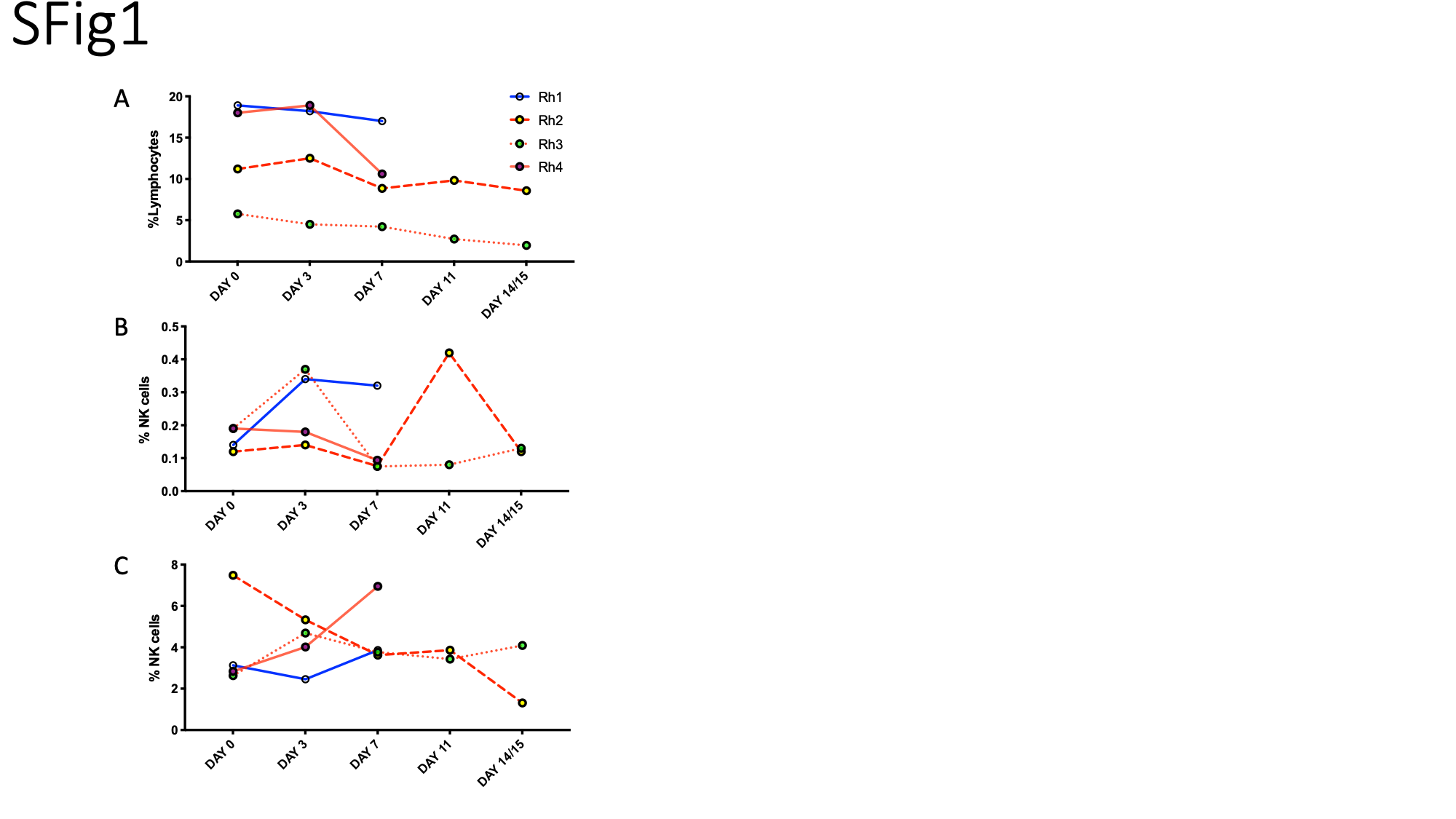

Supplement: Supplementary Figure 1 — Frequencies of peripheral blood CD49a+ NK cells are unchanged during acute SIV infection. (A) Frequencies of NK cells in whole blood of acute-SIV infected animals. Frequencies of (B) CD49a+ or (C) CD49b+ NK cells in the whole blood following acute SIV infection. [file Image_1.tiff]

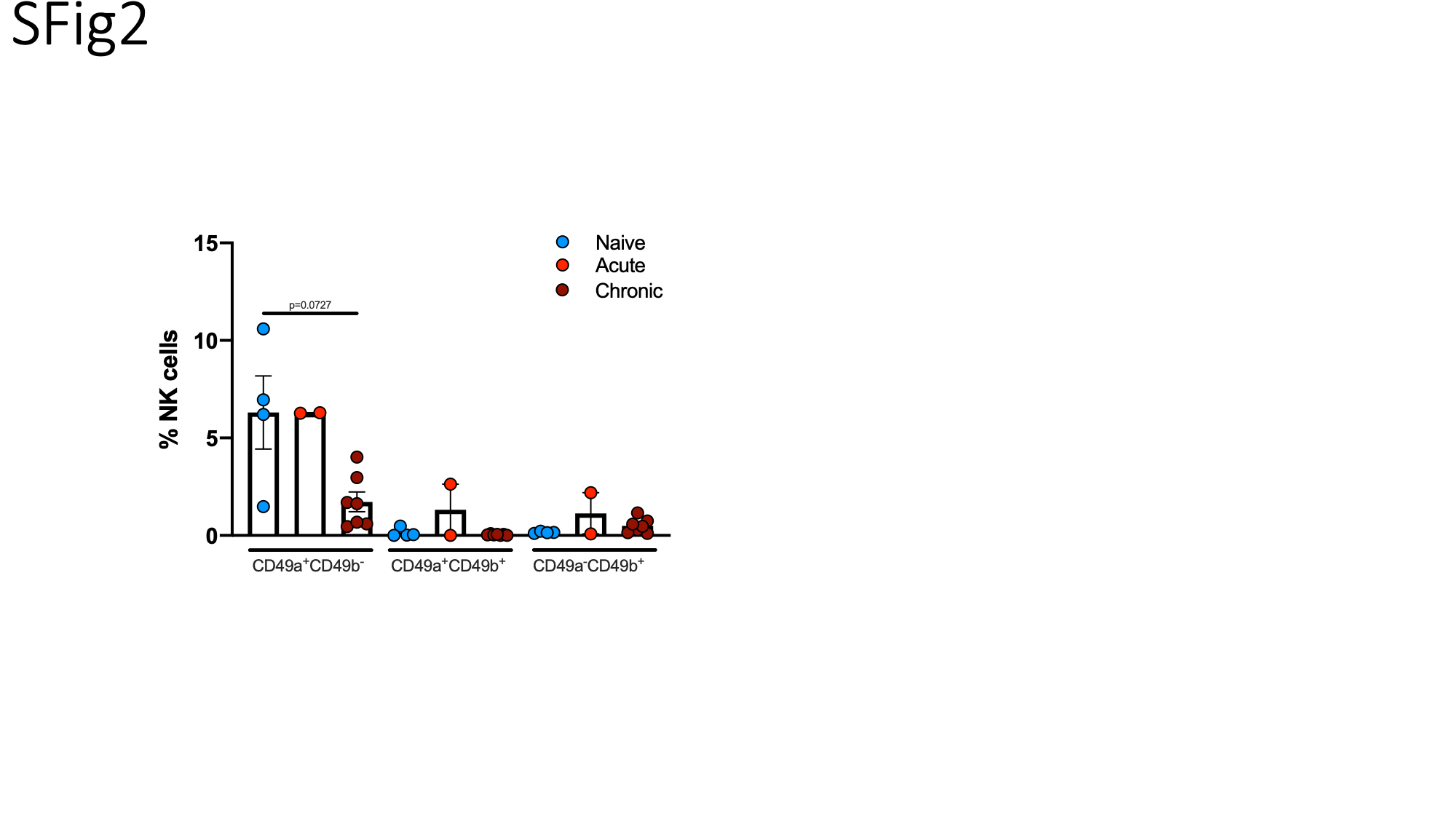

Supplement: Supplementary Figure 2 — Frequencies of CD49a±b± in spleens. Quantification of frequencies of CD49a±CD49b± cells in spleens of naïve (n = 4), acute SIV+ (n = 2), or chronic SHIV+ (n = 7) animals. Mann-Whitney U-test was used to determine statistical significance, *p ≤ 0.05. [file Image_2.tiff]

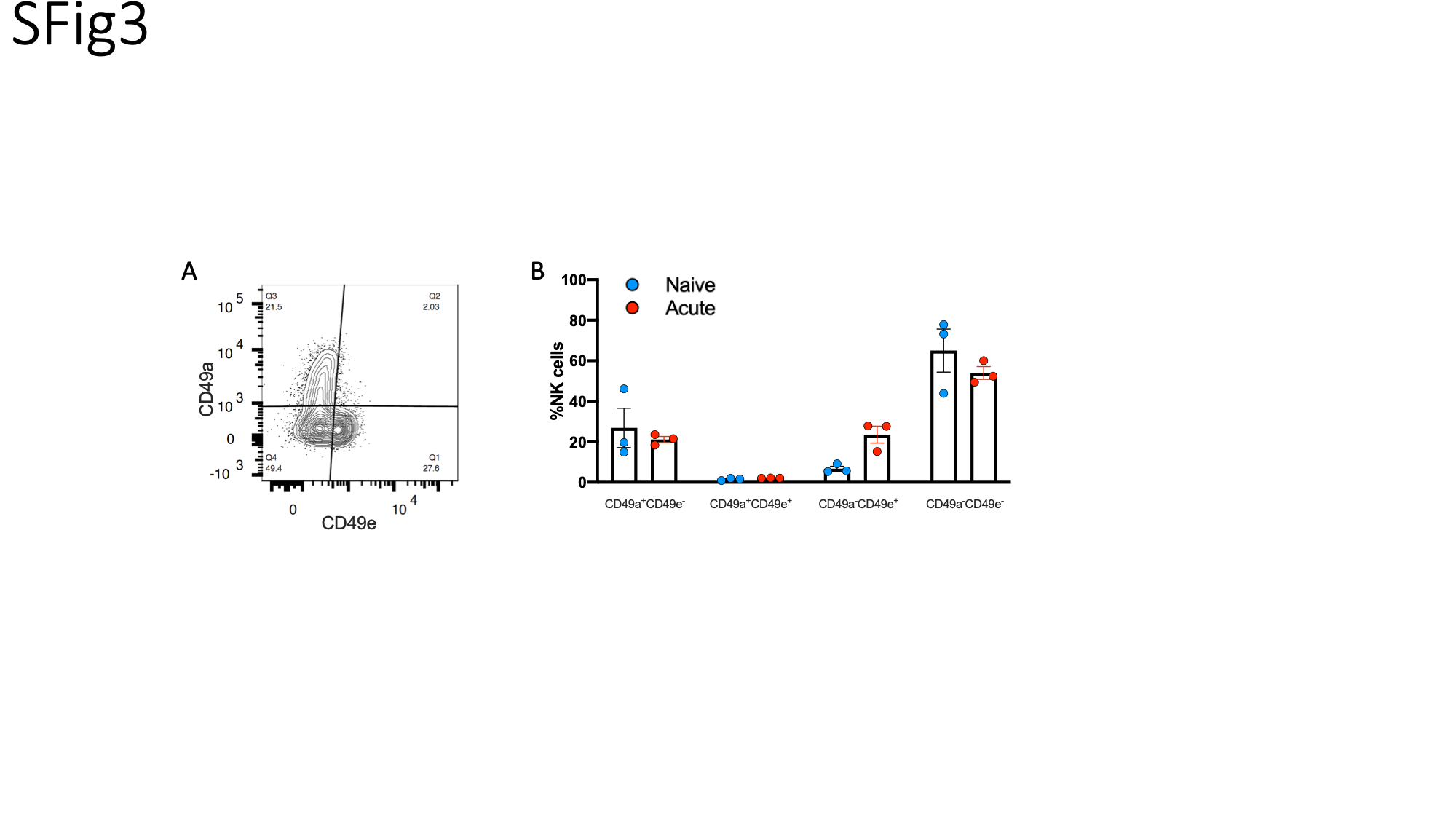

Supplement: Supplementary Figure 3 — Frequencies of CD49a±e± NK cells in livers. (A) Representative flow plot showing identification of CD49a±e± NK cells from liver samples. (B) Quantification of CD49a±e± NK cell populations from livers of naïve (n = 3) and acute SIV+ (n = 3) animals. [file Image_3.tiff]

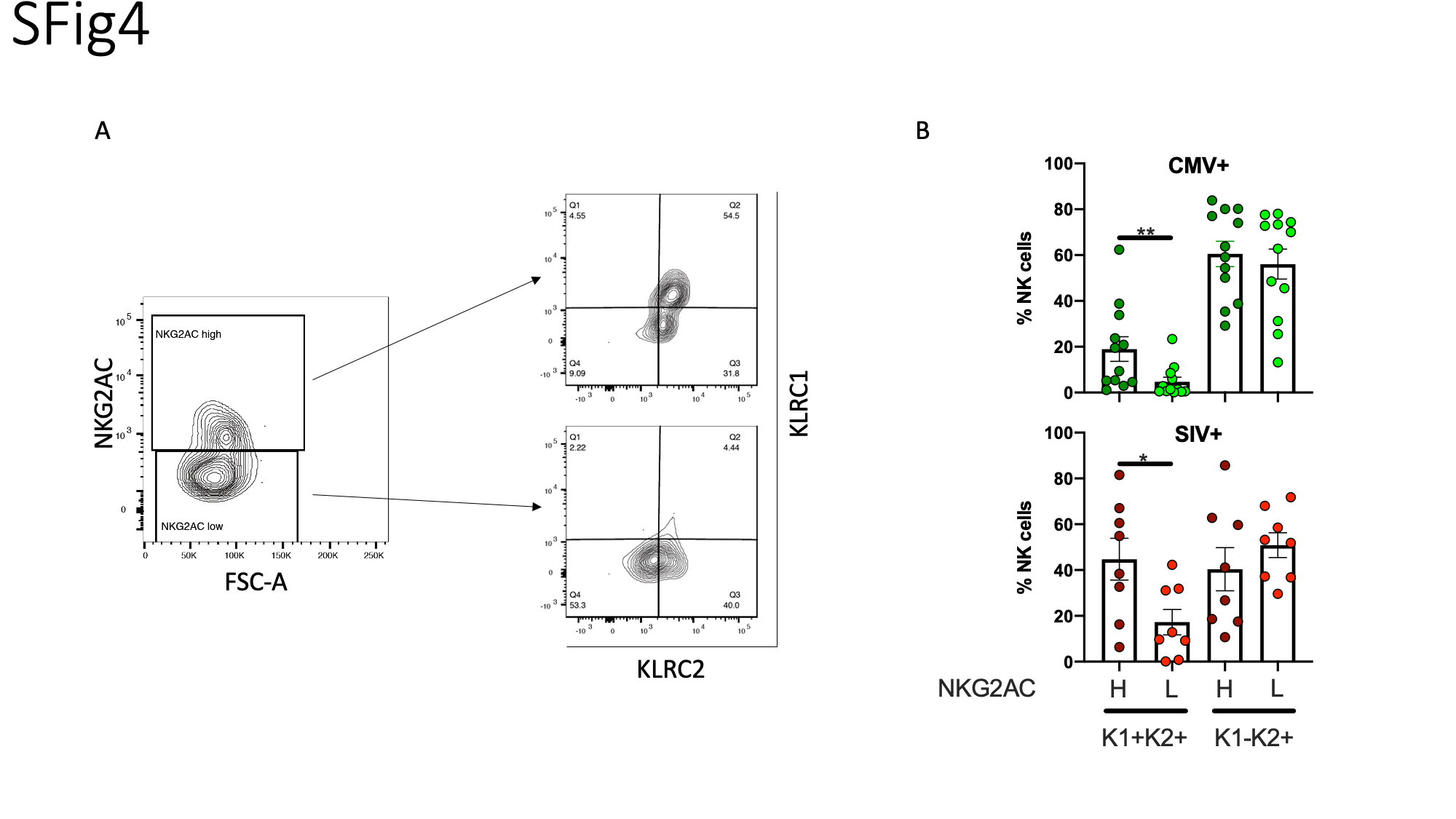

Supplement: Supplementary Figure 4 — NKG2AChigh and NKG2AClow populations exhibit unique KLRC1 and KLRC2 gene expression patterns. (A) Gating strategy showing identification of gene expression of KLRC1 and KLRC2 in NKG2AC high and NKG2AC low populations. (B) Quantification of KLRC1+KLRC2+ (K1+K2+) and KLRC1−KLRC2+ (K1–K2+) populations in NKG2AChigh (H) or NKG2AClow (L) NK cells from CMV+ and SIV-infected animals as per (39). Mann-Whitney U-test was used to determine statistical significance, *p < 0.05, **p < 0.01. [file Image_4.tiff]

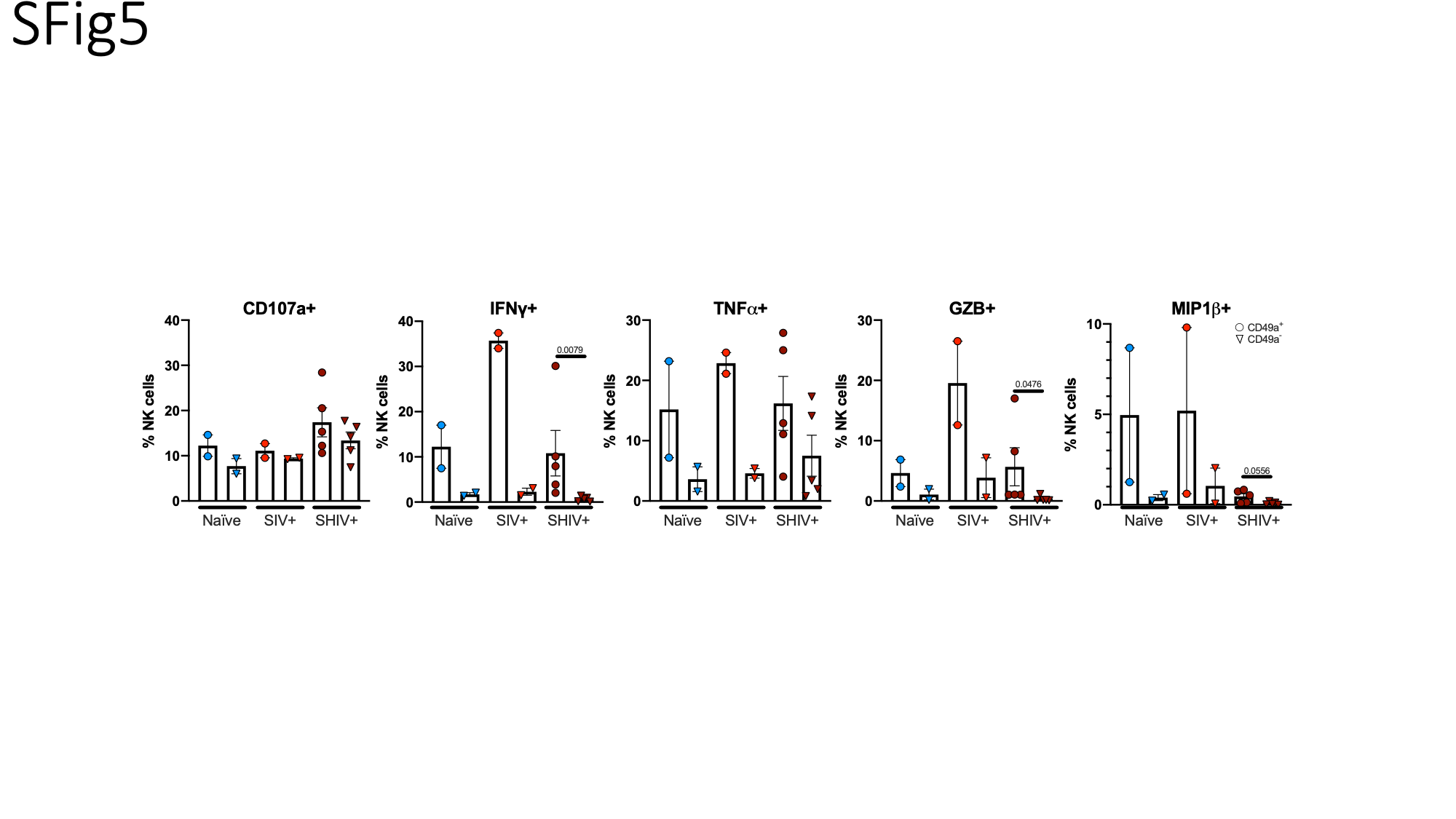

Supplement: Supplementary Figure 5 — Functional characterization of spleen CD49a+ NK cells. (A) Bar graphs showing quantification of CD107a, TNFα, and IFNγ expression in spleen CD49a± NK cells from naïve macaques (n = 2) or macaques acutely infected with SIV (n = 2) or chronically infected with SHIV (n = 5). Wilcoxon test was used to determine statistical significance, *p < 0.05. [file Image_5.tiff]
