# Supplementary material for: Characterization of Rhesus Macaque Liver-Resident CD49a+ NK Cells During Retrovirus Infections
Source: Front Immunol. 2020 Jul 31;11:1676. doi: 10.3389/fimmu.2020.01676 (PMC7411078; doi:10.3389/fimmu.2020.01676)
Supplement: Supplementary Data Sheet 1 — Statistics tables. p-value tables showing comparisons in (A) liver and (B) spleen phenotype data (from Figure 2) as well as (C) permutation testing of polyfunctional populations (from Figure 4B). For (A,B) Wilcox test was used when comparing CD49a+ and CD49a−from the same infection group whereas Mann-Whitney U-test was used when comparing across infection cohorts. p ≤ 0.05 is shown by bold font. 1,000,000 iterations were used in order to carry out the permutation test in (C). p ≤ 0.05 is shown in cells highlighted in pink as generated by SPICE (37). Statistical comparisons between acute and chronic infection groups are not shown for these analyses given the different challenge viruses. [file Data_Sheet_1.PDF]

**A** LIVER

| CD8*α* | Naïve CD49a+ | Naïve CD49a- | SIV+ CD49a+ | SIV+ CD49a- | SHIV+ CD49a+ | SHIV+ CD49a- |
|---|---|---|---|---|---|---|
| Naïve CD49a+ | | *0.875* | 0.9273 | | 0.4127 | |
| Naïve CD49a- | *0.875* | | | 0.9273 | | **0.0159** |
| SIV+ CD49a+ | 0.9273 | | | *0.5781* | | |
| SIV+ CD49a- | | 0.9273 | *0.5781* | | | |
| SHIV+ CD49a+ | 0.4127 | | | | | *0.625* |
| SHIV+ CD49a- | | **0.0159** | | | *0.625* | |

| CD16 | Naïve CD49a+ | Naïve CD49a- | SIV+ CD49a+ | SIV+ CD49a- | SHIV+ CD49a+ | SHIV+ CD49a- |
|---|---|---|---|---|---|---|
| Naïve CD49a+ | | *0.25* | 0.2303 | | 0.1905 | |
| Naïve CD49a- | *0.25* | | | 0.9273 | | >0.9999 |
| SIV+ CD49a+ | 0.2303 | | | ***0.0156*** | | |
| SIV+ CD49a- | | 0.9273 | ***0.0156*** | | | |
| SHIV+ CD49a+ | 0.1905 | | | | | *0.0625* |
| SHIV+ CD49a- | | >0.9999 | | | *0.0625* | |

| CD56 | Naïve CD49a+ | Naïve CD49a- | SIV+ CD49a+ | SIV+ CD49a- | SHIV+ CD49a+ | SHIV+ CD49a- |
|---|---|---|---|---|---|---|
| Naïve CD49a+ | | *0.125* | 0.1636 | | 0.1111 | |
| Naïve CD49a- | *0.125* | | | 0.1091 | | 0.1111 |
| SIV+ CD49a+ | 0.1636 | | | ***0.0156*** | | |
| SIV+ CD49a- | | 0.1091 | ***0.0156*** | | | |
| SHIV+ CD49a+ | 0.1111 | | | | | *0.0625* |
| SHIV+ CD49a- | | 0.1111 | | | *0.0625* | |

| CD62L | Naïve CD49a+ | Naïve CD49a- | SIV+ CD49a+ | SIV+ CD49a- | SHIV+ CD49a+ | SHIV+ CD49a- |
|---|---|---|---|---|---|---|
| Naïve CD49a+ | | *0.125* | 0.6788 | | **0.0317** | |
| Naïve CD49a- | *0.125* | | | 0.1182 | | **0.0079** |
| SIV+ CD49a+ | 0.6788 | | | ***0.0156*** | | |
| SIV+ CD49a- | | 0.1182 | ***0.0156*** | | | |
| SHIV+ CD49a+ | **0.0317** | | | | | *0.1875* |
| SHIV+ CD49a- | | **0.0079** | | | *0.1875* | |

| CD69 | Naïve CD49a+ | Naïve CD49a- | SIV+ CD49a+ | SIV+ CD49a- | SHIV+ CD49a+ | SHIV+ CD49a- |
|---|---|---|---|---|---|---|
| Naïve CD49a+ | | *0.125* | 0.6788 | | 0.5556 | |
| Naïve CD49a- | *0.125* | | | >0.9999 | | **0.0159** |
| SIV+ CD49a+ | 0.6788 | | | ***0.0156*** | | |
| SIV+ CD49a- | | >0.9999 | ***0.0156*** | | | |
| SHIV+ CD49a+ | 0.5556 | | | | | *0.0625* |
| SHIV+ CD49a- | | **0.0159** | | | *0.0625* | |

| CD150 | Naïve CD49a+ | Naïve CD49a- | SIV+ CD49a+ | SIV+ CD49a- | SHIV+ CD49a+ | SHIV+ CD49a- |
|---|---|---|---|---|---|---|
| Naïve CD49a+ | | *0.125* | 0.0727 | | 0.1905 | |
| Naïve CD49a- | *0.125* | | | 0.1091 | | 0.5556 |
| SIV+ CD49a+ | 0.0727 | | | ***0.0156*** | | |
| SIV+ CD49a- | | 0.1091 | ***0.0156*** | | | |
| SHIV+ CD49a+ | 0.1905 | | | | | *0.0625* |
| SHIV+ CD49a- | | 0.5556 | | | *0.0625* | |

| CD336 | Naïve CD49a+ | Naïve CD49a- | SIV+ CD49a+ | SIV+ CD49a- | SHIV+ CD49a+ | SHIV+ CD49a- |
|---|---|---|---|---|---|---|
| Naïve CD49a+ | | *0.125* | >0.9999 | | 0.4127 | |
| Naïve CD49a- | *0.125* | | | 0.5 | | 0.2857 |
| SIV+ CD49a+ | >0.9999 | | | ***0.0156*** | | |
| SIV+ CD49a- | | 0.5 | ***0.0156*** | | | |
| SHIV+ CD49a+ | 0.4127 | | | | | *0.0625* |
| SHIV+ CD49a- | | 0.2857 | | | *0.0625* | |

| CD337 | Naïve CD49a+ | Naïve CD49a- | SIV+ CD49a+ | SIV+ CD49a- | SHIV+ CD49a+ | SHIV+ CD49a- |
|---|---|---|---|---|---|---|
| Naïve CD49a+ | | *0.2* | 0.2303 | | 0.2857 | |
| Naïve CD49a- | *0.25* | | | 0.7879 | | 0.4127 |
| SIV+ CD49a+ | 0.2303 | | | *0.2188* | | |
| SIV+ CD49a- | | 0.7879 | *0.2188* | | | |
| SHIV+ CD49a+ | 0.2857 | | | | | *0.4375* |
| SHIV+ CD49a- | | 0.4127 | | | *0.4375* | |

| CD366 | Naïve CD49a+ | Naïve CD49a- | SIV+ CD49a+ | SIV+ CD49a- | SHIV+ CD49a+ | SHIV+ CD49a- |
|---|---|---|---|---|---|---|
| Naïve CD49a+ | | *0.125* | 0.7879 | | 0.9048 | |
| Naïve CD49a- | *0.125* | | | 0.8333 | | 0.8413 |
| SIV+ CD49a+ | 0.7879 | | | ***0.0156*** | | |
| SIV+ CD49a- | | 0.8333 | ***0.0156*** | | | |
| SHIV+ CD49a+ | 0.9048 | | | | | *0.0625* |
| SHIV+ CD49a- | | 0.8413 | | | *0.0625* | |

| Eomes | Naïve CD49a+ | Naïve CD49a- | SIV+ CD49a+ | SIV+ CD49a- | SHIV+ CD49a+ | SHIV+ CD49a- |
|---|---|---|---|---|---|---|
| Naïve CD49a+ | | *0.125* | **0.0242** | | 0.5556 | |
| Naïve CD49a- | *0.125* | | | 0.1091 | | **0.0159** |
| SIV+ CD49a+ | **0.0242** | | | ***0.0156*** | | |
| SIV+ CD49a- | | 0.1091 | ***0.0156*** | | | |
| SHIV+ CD49a+ | 0.5556 | | | | | *0.0625* |
| SHIV+ CD49a- | | **0.0159** | | | *0.0625* | |

| FcεRI | Naïve CD49a+ | Naïve CD49a- | SIV+ CD49a+ | SIV+ CD49a- | SHIV+ CD49a+ | SHIV+ CD49a- |
|---|---|---|---|---|---|---|
| Naïve CD49a+ | | *0.875* | 0.5273 | | **0.0317** | |
| Naïve CD49a- | *0.875* | | | 0.9273 | | 0.1905 |
| SIV+ CD49a+ | 0.5273 | | | *0.4688* | | |
| SIV+ CD49a- | | 0.9273 | *0.4688* | | | |
| SHIV+ CD49a+ | **0.0317** | | | | | *0.125* |
| SHIV+ CD49a- | | 0.1905 | | | *0.125* | |

**B** SPLEEN

| CD8*α* | Naïve CD49a+ | Naïve CD49a- | SIV+ CD49a+ | SIV+ CD49a- | SHIV+ CD49a+ | SHIV+ CD49a- |
|---|---|---|---|---|---|---|
| Naïve CD49a+ | | *0.125* | 0.1333 | | 0.2303 | |
| Naïve CD49a- | *0.125* | | | 0.1333 | | 0.7879 |
| SIV+ CD49a+ | 0.1333 | | | | | |
| SIV+ CD49a- | | 0.1333 | | | | |
| SHIV+ CD49a+ | 0.2303 | | | | | *0.375* |
| SHIV+ CD49a- | | 0.7879 | | | *0.375* | |

| CD16 | Naïve CD49a+ | Naïve CD49a- | SIV+ CD49a+ | SIV+ CD49a- | SHIV+ CD49a+ | SHIV+ CD49a- |
|---|---|---|---|---|---|---|
| Naïve CD49a+ | | *0.25* | 0.2667 | | 0.3152 | |
| Naïve CD49a- | *0.25* | | | 0.2667 | | 0.5273 |
| SIV+ CD49a+ | 0.2667 | | | | | |
| SIV+ CD49a- | | 0.2667 | | | | |
| SHIV+ CD49a+ | 0.3152 | | | | | *0.1094* |
| SHIV+ CD49a- | | 0.5273 | | | *0.1094* | |

| CD56 | Naïve CD49a+ | Naïve CD49a- | SIV+ CD49a+ | SIV+ CD49a- | SHIV+ CD49a+ | SHIV+ CD49a- |
|---|---|---|---|---|---|---|
| Naïve CD49a+ | | *0.125* | >0.9999 | | 0.1636 | |
| Naïve CD49a- | *0.125* | | | 0.1333 | | 0.9273 |
| SIV+ CD49a+ | >0.9999 | | | | | |
| SIV+ CD49a- | | 0.1333 | | | | |
| SHIV+ CD49a+ | 0.1636 | | | | | ***0.0156*** |
| SHIV+ CD49a- | | 0.9273 | | | ***0.0156*** | |

| CD336 | Naïve CD49a+ | Naïve CD49a- | SIV+ CD49a+ | SIV+ CD49a- | SHIV+ CD49a+ | SHIV+ CD49a- |
|---|---|---|---|---|---|---|
| Naïve CD49a+ | | *0.125* | 0.1333 | | 0.4939 | |
| Naïve CD49a- | *0.125* | | | 0.5333 | | 0.2303 |
| SIV+ CD49a+ | 0.1333 | | | | | |
| SIV+ CD49a- | | 0.5333 | | | | |
| SHIV+ CD49a+ | 0.4939 | | | | | ***0.0781*** |
| SHIV+ CD49a- | | 0.2303 | | | ***0.0781*** | |

| CD337 | Naïve CD49a+ | Naïve CD49a- | SIV+ CD49a+ | SIV+ CD49a- | SHIV+ CD49a+ | SHIV+ CD49a- |
|---|---|---|---|---|---|---|
| Naïve CD49a+ | | *>0.9999* | 0.1333 | | 0.8818 | |
| Naïve CD49a- | *>0.9999* | | | 0.2667 | | 0.5273 |
| SIV+ CD49a+ | 0.1333 | | | | | |
| SIV+ CD49a- | | 0.2667 | | | | |
| SHIV+ CD49a+ | 0.8818 | | | | | *0.1562* |
| SHIV+ CD49a- | | 0.5273 | | | *0.1562* | |

| CD366 | Naïve CD49a+ | Naïve CD49a- | SIV+ CD49a+ | SIV+ CD49a- | SHIV+ CD49a+ | SHIV+ CD49a- |
|---|---|---|---|---|---|---|
| Naïve CD49a+ | | *0.25* | 0.1333 | | 0.4939 | |
| Naïve CD49a- | *0.25* | | | 0.1333 | | 0.7879 |
| SIV+ CD49a+ | 0.1333 | | | | | |
| SIV+ CD49a- | | 0.1333 | | | | |
| SHIV+ CD49a+ | 0.4939 | | | | | *0.1094* |
| SHIV+ CD49a- | | 0.7879 | | | *0.1094* | |

| Eomes | Naïve CD49a+ | Naïve CD49a- | SIV+ CD49a+ | SIV+ CD49a- | SHIV+ CD49a+ | SHIV+ CD49a- |
|---|---|---|---|---|---|---|
| Naïve CD49a+ | | *0.75* | 0.5333 | | 0.3152 | |
| Naïve CD49a- | *0.75* | | | 0.5333 | | 0.3152 |
| SIV+ CD49a+ | 0.5333 | | | | | |
| SIV+ CD49a- | | 0.5333 | | | | |
| SHIV+ CD49a+ | 0.3152 | | | | | ***0.0156*** |
| SHIV+ CD49a- | | 0.3152 | | | ***0.0156*** | |

| Statistical Analysis Key |
|---|
| Mann-Whitney U |
| *Wilcoxon* |
| ***p < 0.05*** |

| HLA-DR | Naïve CD49a+ | Naïve CD49a- | SIV+ CD49a+ | SIV+ CD49a- | SHIV+ CD49a+ | SHIV+ CD49a- |
|---|---|---|---|---|---|---|
| Naïve CD49a+ | | *0.625* | 0.3152 | | 0.5556 | |
| Naïve CD49a- | *0.625* | | | 0.0727 | | 0.1111 |
| SIV+ CD49a+ | 0.3152 | | | *>0.9999* | | |
| SIV+ CD49a- | | 0.0727 | *>0.9999* | | | |
| SHIV+ CD49a+ | 0.5556 | | | | | *0.125* |
| SHIV+ CD49a- | | 0.1111 | | | *0.125* | |

| HLA-DR | Naïve CD49a+ | Naïve CD49a- | SIV+ CD49a+ | SIV+ CD49a- | SHIV+ CD49a+ | SHIV+ CD49a- |
|---|---|---|---|---|---|---|
| Naïve CD49a+ | | *0.375* | 0.1333 | | 0.1636 | |
| Naïve CD49a- | *0.375* | | | 0.5333 | | 0.3394 |
| SIV+ CD49a+ | 0.1333 | | | | | |
| SIV+ CD49a- | | 0.5333 | | | | |
| SHIV+ CD49a+ | 0.1636 | | | | | *0.0781* |
| SHIV+ CD49a- | | 0.3394 | | | *0.0781* | |

| NKG2AC high | Naïve CD49a+ | Naïve CD49a- | SIV+ CD49a+ | SIV+ CD49a- | SHIV+ CD49a+ | SHIV+ CD49a- |
|---|---|---|---|---|---|---|
| Naïve CD49a+ | | *0.875* | >0.9999 | | 0.1905 | |
| Naïve CD49a- | *0.875* | | | 0.8212 | | 0.5556 |
| SIV+ CD49a+ | >0.9999 | | | *0.1094* | | |
| SIV+ CD49a- | | 0.8212 | *0.1094* | | | |
| SHIV+ CD49a+ | 0.1905 | | | | | *0.0625* |
| SHIV+ CD49a- | | 0.5556 | | | *0.0625* | |

| NKG2AC high | Naïve CD49a+ | Naïve CD49a- | SIV+ CD49a+ | SIV+ CD49a- | SHIV+ CD49a+ | SHIV+ CD49a- |
|---|---|---|---|---|---|---|
| Naïve CD49a+ | | *0.125* | 0.1333 | | **0.0061** | |
| Naïve CD49a- | *0.125* | | | 0.5333 | | 0.0727 |
| SIV+ CD49a+ | 0.1333 | | | | | |
| SIV+ CD49a- | | 0.5333 | | | | |
| SHIV+ CD49a+ | **0.0061** | | | | | ***0.0156*** |
| SHIV+ CD49a- | | 0.0727 | | | ***0.0156*** | |

| NKG2AC low | Naïve CD49a+ | Naïve CD49a- | SIV+ CD49a+ | SIV+ CD49a- | SHIV+ CD49a+ | SHIV+ CD49a- |
|---|---|---|---|---|---|---|
| Naïve CD49a+ | | *0.875* | >0.9999 | | 0.1905 | |
| Naïve CD49a- | *0.875* | | | 0.7879 | | 0.5556 |
| SIV+ CD49a+ | >0.9999 | | | *0.0781* | | |
| SIV+ CD49a- | | 0.7879 | *0.0781* | | | |
| SHIV+ CD49a+ | 0.1905 | | | | | *0.0625* |
| SHIV+ CD49a- | | 0.5556 | | | *0.0625* | |

| NKG2AC low | Naïve CD49a+ | Naïve CD49a- | SIV+ CD49a+ | SIV+ CD49a- | SHIV+ CD49a+ | SHIV+ CD49a- |
|---|---|---|---|---|---|---|
| Naïve CD49a+ | | ***0.0286*** | 0.1333 | | **0.0061** | |
| Naïve CD49a- | ***0.0286*** | | | 0.5333 | | 0.0727 |
| SIV+ CD49a+ | 0.1333 | | | | | |
| SIV+ CD49a- | | 0.5333 | | | | |
| SHIV+ CD49a+ | **0.0061** | | | | | ***0.0156*** |
| SHIV+ CD49a- | | 0.0727 | | | ***0.0156*** | |

| PD-1 | Naïve CD49a+ | Naïve CD49a- | SIV+ CD49a+ | SIV+ CD49a- | SHIV+ CD49a+ | SHIV+ CD49a- |
|---|---|---|---|---|---|---|
| Naïve CD49a+ | | *0.625* | **0.0424** | | 0.1905 | |
| Naïve CD49a- | *0.625* | | | **0.0424** | | 0.2857 |
| SIV+ CD49a+ | **0.0424** | | | *>0.9999* | | |
| SIV+ CD49a- | | **0.0424** | *>0.9999* | | | |
| SHIV+ CD49a+ | 0.1905 | | | | | *0.8125* |
| SHIV+ CD49a- | | 0.2857 | | | *0.8125* | |

| Syk | Naïve CD49a+ | Naïve CD49a- | SIV+ CD49a+ | SIV+ CD49a- | SHIV+ CD49a+ | SHIV+ CD49a- |
|---|---|---|---|---|---|---|
| Naïve CD49a+ | | *0.375* | 0.6848 | | **0.0159** | |
| Naïve CD49a- | *0.375* | | | >0.9999 | | **0.0159** |
| SIV+ CD49a+ | 0.6848 | | | *0.2188* | | |
| SIV+ CD49a- | | >0.9999 | *0.2188* | | | |
| SHIV+ CD49a+ | **0.0159** | | | | | *>0.9999* |
| SHIV+ CD49a- | | **0.0159** | | | *>0.9999* | |

| T-bet | Naïve CD49a+ | Naïve CD49a- | SIV+ CD49a+ | SIV+ CD49a- | SHIV+ CD49a+ | SHIV+ CD49a- |
|---|---|---|---|---|---|---|
| Naïve CD49a+ | | *0.25* | 0.5273 | | 0.7302 | |
| Naïve CD49a- | *0.25* | | | 0.1636 | | 0.9048 |
| SIV+ CD49a+ | 0.5273 | | | *0.1094* | | |
| SIV+ CD49a- | | 0.1626 | *0.1094* | | | |
| SHIV+ CD49a+ | 0.7302 | | | | | *0.0625* |
| SHIV+ CD49a- | | 0.9048 | | | *0.0625* | |

| T-bet | Naïve CD49a+ | Naïve CD49a- | SIV+ CD49a+ | SIV+ CD49a- | SHIV+ CD49a+ | SHIV+ CD49a- |
|---|---|---|---|---|---|---|
| Naïve CD49a+ | | *0.125* | 0.2667 | | 0.8212 | |
| Naïve CD49a- | *0.125* | | | 0.1333 | | 0.7879 |
| SIV+ CD49a+ | 0.2667 | | | | | |
| SIV+ CD49a- | | 0.1333 | | | | |
| SHIV+ CD49a+ | 0.8212 | | | | | ***0.0156*** |
| SHIV+ CD49a- | | 0.7879 | | | ***0.0156*** | |

**C**

| | | Naïve | | Acute | | Chronic | |
|---|---|---|---|---|---|---|---|
| | | CD49a+ | CD49a - | CD49a+ | CD49a - | CD49a+ | CD49a - |
| Naïve | CD49a+ | | 0.4739 | 0.3067 | 0.8678 | 0.0492 | 0.0745 |
| | CD49a - | 0.4739 | | 0.0721 | 0.9186 | 0.0163 | 0.0417 |
| Acute | CD49a+ | 0.3067 | 0.0721 | | 0.2616 | 0.3538 | 0.2336 |
| | CD49a - | 0.8678 | 0.9186 | 0.2616 | | 0.1079 | 0.2257 |
| Chronic | CD49a+ | 0.0492 | 0.0163 | 0.3538 | 0.1079 | | 0.5265 |
| | CD49a - | 0.0745 | 0.0417 | 0.2336 | 0.2257 | 0.5265 | |
